# Projecting COVID-19 Mortality as States Relax Nonpharmacologic Interventions

**DOI:** 10.1001/jamahealthforum.2022.0760

**Published:** 2022-04-01

**Authors:** Benjamin P. Linas, Jade Xiao, Ozden O. Dalgic, Peter P. Mueller, Madeline Adee, Alec Aaron, Turgay Ayer, Jagpreet Chhatwal

**Affiliations:** 1 Boston Medical Center, Boston, Massachusetts; 2 Boston University Schools of Medicine and Public Health, Boston, Massachusetts; 3 H. Milton Stewart School of Industrial and Systems Engineering, Georgia Institute of Technology, Atlanta; 4 Value Analytics Labs, Boston, Massachusetts; 5 Massachusetts General Hospital Institute for Technology Assessment, Boston; 6 Harvard Medical School, Boston, Massachusetts

## Abstract

**Importance:**

A key question for policy makers and the public is what to expect from the COVID-19 pandemic going forward as states lift nonpharmacologic interventions (NPIs), such as indoor mask mandates, to prevent COVID-19 transmission.

**Objective:**

To project COVID-19 deaths between March 1, 2022, and December 31, 2022, in each of the 50 US states, District of Columbia, and Puerto Rico assuming different dates of lifting of mask mandates and NPIs.

**Design, Setting, and Participants:**

This simulation modeling study used the COVID-19 Policy Simulator compartmental model to project COVID-19 deaths from March 1, 2022, to December 31, 2022, using simulated populations in the 50 US states, District of Columbia, and Puerto Rico. Projected current epidemiologic trends for each state until December 31, 2022, assuming the current pace of vaccination is maintained into the future and modeling different dates of lifting NPIs.

**Exposures:**

Date of lifting statewide NPI mandates as March 1, April 1, May 1, June 1, or July 1, 2022.

**Main Outcomes and Measures:**

Projected COVID-19 incident deaths from March to December 2022.

**Results:**

With the high transmissibility of current circulating SARS-CoV-2 variants, the simulated lifting of NPIs in March 2022 was associated with resurgences of COVID-19 deaths in nearly every state. In comparison, delaying by even 1 month to lift NPIs in April 2022 was estimated to mitigate the amplitude of the surge. For most states, however, no amount of delay was estimated to be sufficient to prevent a surge in deaths completely. The primary factor associated with recurrent epidemics in the simulation was the assumed high effective reproduction number of unmitigated viral transmission. With a lower level of transmissibility similar to those of the ancestral strains, the model estimated that most states could remove NPIs in March 2022 and likely not see recurrent surges.

**Conclusions and Relevance:**

This simulation study estimated that the SARS-CoV-2 virus would likely continue to take a major toll in the US, even as cases continued to decrease. Because of the high transmissibility of the recent Delta and Omicron variants, premature lifting of NPIs could pose a substantial threat of rebounding surges in morbidity and mortality. At the same time, continued delay in lifting NPIs may not prevent future surges.

## Key Points

**Question:**

What is the expected trend in COVID-19 mortality if US states were to lift nonpharmacologic interventions (NPIs) at different times over the remainder of 2022?

**Findings:**

In this simulation modeling study, lifting NPIs was likely to result in rebounding epidemics regardless of the delay in lifting. The degree of population-level immunity was associated with the size of the rebounding peak in incident deaths.

**Meaning:**

This simulation study found no path to the end of the COVID-19 pandemic that avoided difficult trade-offs between prolonged NPIs and increased COVID-19 mortality following their removal.

## Teaser

This simulation modeling study projects COVID-19 deaths between March 1, 2022, and December 31, 2022, in each of the 50 US states, District of Columbia, and Puerto Rico assuming different dates of lifting of mask mandates and nonpharmacologic interventions.

## Introduction

The emergency authorization and dissemination of SARS-CoV-2 vaccines starting in late 2020 fundamentally changed the epidemiology of the COVID-19 pandemic. Leading up to the summer of 2021, nearly every state enjoyed falling case rates while simultaneously relaxing nonpharmacologic interventions (NPIs), such as mask mandates and restrictions on social gatherings. This dissociation of social mobility and COVID-19 case rates was a categorical shift that implied a pending end to the pandemic. Unfortunately, around this time, the Delta variant entered circulation in the US and quickly became the dominant SARS-CoV-2 strain. The period of falling case rates was ended by the “fourth wave,” even in states that had achieved relatively high levels of vaccination. Next, even as the Delta variant was still spreading rapidly, the Omicron surge arrived, driving case rates to the highest levels seen in the pandemic and forcing some jurisdictions to reinstate mitigation measures.

The return of NPIs and ongoing need to integrate COVID-19 risk into everyday decision-making have greatly added to pandemic fatigue in the US. Now, as the Omicron wave begins to recede, many states are once again lifting mandatory NPIs, including indoor capacity limits and guidance on social distancing. In particular, local decision makers face the difficult decision of when to lift mask mandates. One of the most important questions currently on the minds of citizens, public health officials, and policy makers is: when can we safely lift restrictions?

We used the COVID-19 Policy Simulator,^1^ a compartmental model of SARS-CoV-2 transmission and COVID-19 disease in the US, to project rates of hospitalization and death over the course of the 2022 calendar year assuming different dates of lifting NPIs.

## Methods

### Model Overview

Our model is an extension of the traditional SEIR model, which partitions a population into compartments representing mutually exclusive disease states: susceptible, exposed, infected, recovered, and deceased. In this model, the flow of people between compartments was assumed to obey a system of deterministic ordinary differential equations. The time step was set 1 day to be compatible with data sources reporting daily data. The model was calibrated to historical trends in daily incident deaths up to February 20, 2022. The Table displays the values and data sources for select model parameters. A full model specification is provided in the eAppendix in the Supplement. Where applicable, we followed the Consolidated Health Economic Evaluation Reporting Standards (CHEERS) framework for communicating results. The study was exempt from institutional review board review because it used only publicly available data and was not human participants research.

**Table.** Values of Select Parameters Used in the COVID-19 Policy Simulator Model

Parameter	Estimate	Notes	Reference
**Fixed parameters**			
Size of the subpopulations <65 y (lower risk) and ≥65 y (higher risk)	State dependent	NA	US Census Bureau,^2^ 2021
Contact matrix			
LL	0.93	Aggregate columns and rows into age groups <65 y and ≥65 y, then normalize so that rows sum to 1.	Prem et al,^3^ 2017
LH	0.07		
HL	0.48		
HH	0.52		
Period, d			
Latent	5.5	NA	Xin et al,^4^ 2019
Infectious	10	NA	Byrne et al,^5^ 2020
Mean (exponentially distributed) duration of natural and vaccine-conferred immunity, mo	16	NA	Townsend et al,^6^ 2021
Effective reproduction number when all NPIs are removed	5.0	NA	Liu and Rocklöv,^7^ 2021
**Calibrated parameters**			
Time-varying effective reproduction number	0.5-6.0	Widely varying by location and SARS-CoV-2 variant	Liu and Rocklöv,^7^ 2021
Initial number of infectious people at the start of the simulation (March 15, 2020)	100-10 000	Calibrated and divided proportionally into the low-risk and high-risk groups	NA
**Variant-dependent parameters (see eAppendix in the Supplement for derivation and changes associated with the Delta and Omicron variants)**			
Baseline, %			
IFR of the low-risk/high-risk group	0.1/3.0	These values chosen to approximate the CDC’s estimated total infections^8^	Based on this meta-analysis^9^
Reduction in susceptibility to infection after the 1st/2nd vaccine dose	46/92	NA	Dagan et al,^10^ 2021
Reduction in IFR after the 1st/2nd vaccine dose	48/37	It is the conditional probability of death that is higher after the second dose than after the first dose. If a fully vaccinated individual contracts a breakthrough infection despite 92% reduction in susceptibility, it is plausible that they are particularly vulnerable and have a smaller reduction in mortality risk conditional on infection compared with a partially vaccinated individual who contracts a breakthrough infection.	Dagan et al,^10^ 2021

Abbreviations: CDC, Centers for Disease Control and Prevention; IFR, infection fatality rate; HH, high-high risk; HL, high-low risk; LH, low-high risk, LL, low-low risk; NA, not applicable; NPI, nonpharmacologic intervention.

### Age Stratification

We stratified the population into 2 age groups: younger than 65 years (lower risk) and 65 years and older (higher risk), assuming that the size of these subpopulations was constant over the simulation period. Age stratification allowed the model to capture differential vaccination trends and COVID-19 mortality between the age groups.

### Mortality

We estimated COVID-19–associated mortality using an infection fatality rate (IFR). The IFR is age specific: 0.5% for the younger than 65 years age group and 3.0% for the 65 years and older age group.

### Vaccination

To reflect 2-dose administration guidelines of the COVID-19 messenger RNA vaccines, we stratified the disease states by vaccination status: 0 doses (unvaccinated), 1 dose (partially vaccinated), and 2 doses (fully vaccinated). The third vaccine, the viral vector vaccine, approved for a single-dose regimen, was omitted from the model owing to its accounting for only 3.7% of all administered doses in the US as of June 2021.^11^ Because there are no data on vaccination status at the time of infection, we assumed doses were allocated proportionally to the susceptible and recovered compartments over the historical time horizon. The vaccine reduces both susceptibility to infection and mortality risk. After the first and second vaccine doses, the probability of contracting the virus was reduced by 46% and 92%, respectively; similarly, the IFR was reduced by 48% and 37%, respectively (see eAppendix in the Supplement for derivation). Note that it is the conditional probability of death that is higher after the second dose than after the first dose, such that the overall reduction in COVID-19 mortality is 72% and 95%, respectively. Vaccine effectiveness was assumed to decrease as the Delta and Omicron variants enter circulation. We defined *effective immunity* as the sum of the proportion of the populations in the unvaccinated, partially vaccinated, and fully vaccinated susceptible states, weighted by their susceptibility to infection.

### Transmission

For a susceptible individual, the rate of exposure to the virus was dependent on the individual’s risk group, vaccination status, time-varying effective reproduction number, and the size of the infected subpopulation. We estimated an age-stratified matrix of contact patterns among and between the age groups (Table).

### Waning Immunity

An individual who has recovered from natural infection would experience a period of natural immunity before transitioning back into the susceptible state. A fully vaccinated susceptible individual would be protected for the duration of vaccine-conferred immunity before transitioning back into the partially susceptible state. Finally, because individuals with natural immunity who are subsequently vaccinated have been reported to exhibit “unusually potent immune responses,”^12^ a fully vaccinated recovered individual was assumed to possess 2 “layers” of immunity, shedding first their natural immunity then their vaccine-conferred immunity. At present, there are no certain estimates of the mean duration of natural and vaccine-conferred immunity. We used the results of a study that examined the immune responses to evolutionarily similar viruses to estimate their time to reinfection under endemic conditions.^6^ Reinfection by endemic SARS-CoV-2 was expected to occur between 3 months and 5 years after peak antibody response, with a median of 16 months.

### Booster Shots

It was assumed that, once vaccinated, an individual would never shed their immunity completely (within the time frame of the simulation), and a fully vaccinated individual who has shed their vaccine-conferred immunity would be indistinguishable from a partially vaccinated individual. Thus, the model differentiated between the subpopulation that was willing to receive booster shots and the subpopulation that was unwilling to be vaccinated. Fully vaccinated individuals would wane into the partially vaccinated state and would be “boosted” back into the fully vaccinated state.

### Scenario Analysis

We projected epidemiologic trends assuming that current rates of infection and vaccination would continue until the date of lifting NPIs, which are the beginning of each calendar month from March to July 2022. We allowed the most recent calibrated value of the effective reproduction number to persist until the lifting date, after which it was increased to the assumed value of 5.0, similar to the basic reproduction number of the Delta variant, representing unmitigated transmission of the virus. We also present projections assuming a lower value of the effective reproduction number of 3.0, similar to the transmissibility of the ancestral strains.^7^

### Outcomes and Statistical Analysis

The primary outcome was projections of COVID-19 incident deaths during the remainder of the 2022 calendar year in the 50 US states, the District of Columbia, and Puerto Rico. In addition, we calculated the Spearman rank correlation coefficient as a measure of the correlation between population-level immunity at the time of lifting NPIs and the height of the rebounding surges in COVID-19 deaths. Analyses were performed in February and March 2022 using R, version 4.0.3 (R Foundation for Statistical Computing).

## Results

Simulation outcomes indicate that in almost every state, lifting NPIs in 2022 would lead to a substantial rebound in COVID-19 deaths, with peak incident deaths rivaling those seen at the peak of the Omicron surge if lifting occurred in March 2022. Beyond that, however, delaying lifting would not benefit every state equally. In California, Montana, North Carolina, and Oregon, incremental 1-month delays in lifting was estimated to mitigate the amplitude of the rebounding peak in incident deaths. In contrast, the predicted peaks in Florida, Illinois, Michigan, Tennessee, and Washington are similar in size regardless of the timing of lifting, indicating that prolonging restrictions would not meaningfully reduce the disease burden. Moreover, in Massachusetts, New Jersey, New York, and Ohio, delaying lifting was estimated to increase the subsequent peak in incident deaths. Panel A in the Figure presents projections for select states in each group of qualitative outcomes. A complete set of state projections can be found in the eAppendix in the Supplement.

**Figure.**
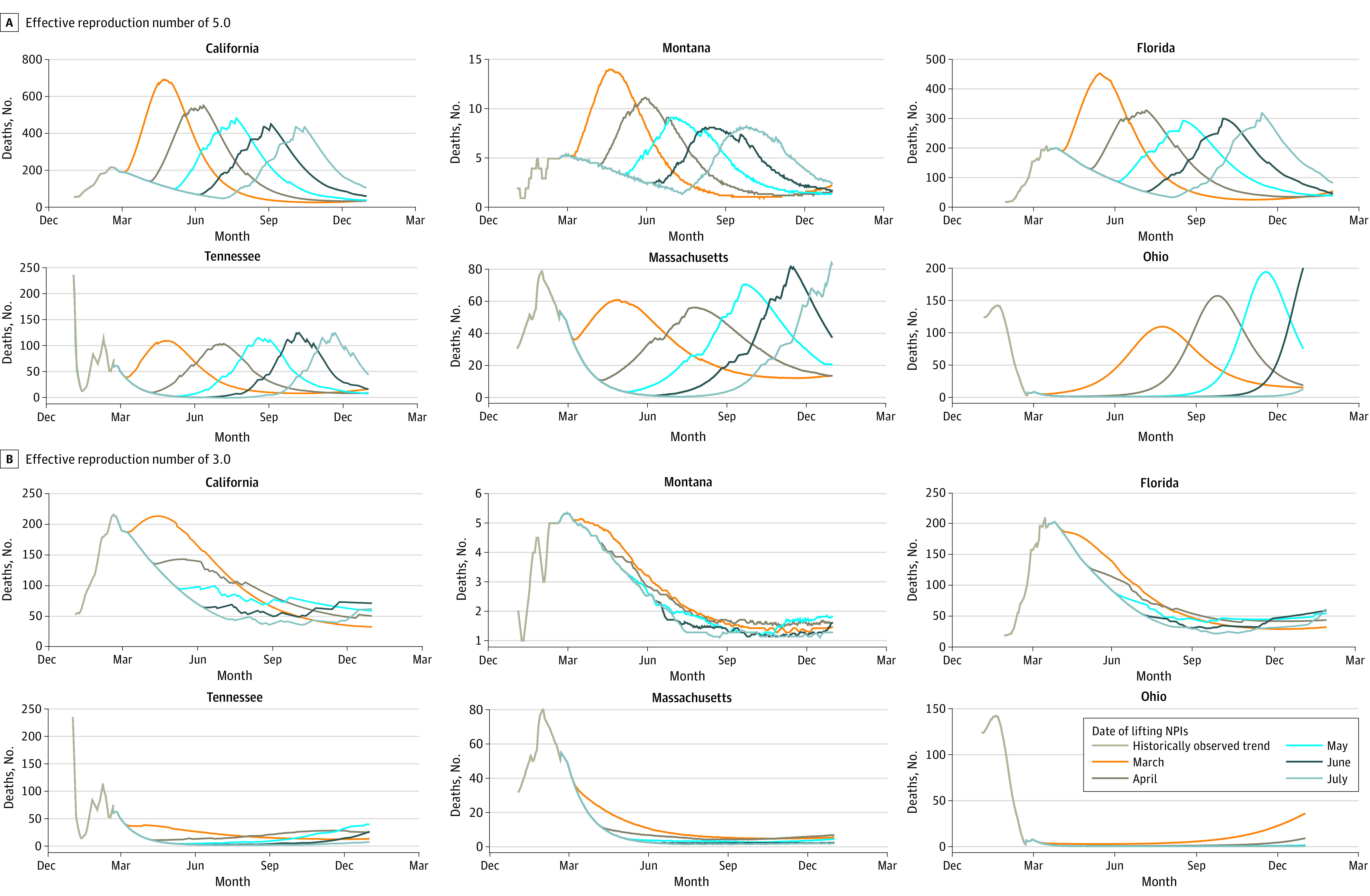
Projected COVID-19 Incident Deaths Model-based projections of COVID-19 deaths in 2022 following the lifting of nonpharmacologic interventions in California, Montana, Florida, Tennessee, Massachusetts, and Ohio, assuming an effective reproduction number of 5.0 (A) and 3.0 (B).

Heterogeneity in the magnitude of the resurgent epidemic across states was driven by the assumption of a single value of the effective reproduction number when no NPIs were in place. It is plausible that smaller states would never reach the level of transmission implied by an effective reproduction number of 5.0 owing to lower population density and activity; hence, the results may be overestimating the severity of their outlook. Panel B in the Figure presents projections with a lower reproduction number of 3.0, in which case the majority of states could lift restrictions with minimal COVID-19 repercussions.

Heterogeneity in response was also associated with differential levels of immunity from both natural infection and vaccination. The combination of waning immunity and falling rates of infection and vaccination means that the net change in population-level immunity would eventually become negative, such that longer delays in lifting could be associated with larger rebounding epidemics. Therefore, current levels of immunity are crucial determinants of the outcomes of returning to higher levels of transmission. We define *effective immunity* as the sum of the proportion of the populations in the unvaccinated, partially vaccinated, and fully vaccinated susceptible states, weighted by their susceptibility to infection. The Spearman rank correlation coefficient between peak incident deaths (as a percentage of the total population) following the lifting of restrictions on March 1, 2022, and effective immunity on February 20, 2022, was −0.88 (*P* < 2.2^−16^). This highly significant and strongly negative correlation suggests that immunity to infection may be associated with a reduction in the severity of the ensuing epidemic following the lifting of NPIs.

## Discussion

We used the COVID-19 Policy Simulator to forecast the number of COVID-19 deaths in each of the 50 US states, the District of Columbia, and Puerto Rico through the 2022 calendar year pending relaxation of NPIs. This analysis could potentially aid state public health officials in evaluating the costs and benefits of lifting NPIs and the timing thereof.

The analysis demonstrates the importance of the timing of lifting NPIs. Premature lifting was estimated to result in recurrent epidemic surges in every almost state. At the same time, a delay of even 1 month was estimated to result in marked reductions to the peak of the mortality curve and the burden on US hospitals. Unfortunately, in most states, no critical moment was identified after which it would be possible to lift NPIs without expecting to see a rebounding surge in deaths. The message that there is no “magic moment” to lift restrictions is important for both sides of the current masking debates in the US. Those opposed to mask mandates should recognize the adverse health outcomes related to relaxing transmission mitigation measures. Any argument to remove such restrictions must address the trade-off and explicitly argue for lifting restrictions within a cost–benefit framework examining the cost of restrictions vs the cost of COVID-19 mortality. At the same time, those who favor maintaining NPIs must recognize that “just a little longer” will not suffice. There is likely no amount of additional waiting time in any state after which removing NPIs will not lead to a rise in morbidity and mortality. The same logic and goals that drive mitigation today will persist, emphasizing the need for mitigation in the future.

A difficult trade-off lies on the horizon. The decision need not be made today, and there is ample evidence that a March 2022 lifting date would have been too soon in many states. However, whenever states do remove NPIs, they will face the same difficult decision regarding the trade-off between increased COVID-19 mortality and the freedoms of returning to a prepandemic norm.

We also estimate that the highly transmissible Delta and Omicron variants will likely continue to take a major toll on the US. The simulations reveal that it is the high transmissibility of these recent variants that sustains the pandemic. With a lower level of transmission similar to that of the ancestral strains, the burden of rebounding morbidity and mortality would be substantially lower. Were this the case, it would likely be possible to remove NPIs at the beginning of the second quarter of 2022.

### Limitations

This study has important limitations. First, we use simulation modeling, which includes all caveats that past performance does not ensure future performance. The COVID-19 Policy Simulator closely replicates historical trends in COVID-19 cases and deaths in all states, but it cannot forecast trends introduced by entirely new dynamics, such as new SARS-CoV-2 variants. Second, the true level of transmission following the lifting of NPIs is uncertain but is obviously a key driver of the outcomes of the analysis. We have presented outcomes with a pessimistic and an optimistic value of the effective reproduction number to allow readers to make their own assessments. Third, the model does not incorporate interstate travel. Predictions may be biased in states that typically experience a high level of travel from other states that have differing levels of COVID-19 cases. Fourth, the model assumes that when NPIs are removed, the virus returns to the level of transmissibility expected in the complete absence of mitigation measures. In reality, individuals may voluntarily continue to wear masks and practice social distancing, which could mitigate the severity of rebounding epidemics.

## Conclusions

This study used simulation modeling to project COVID-19 deaths in the each of the 50 US states, the District of Columbia, and Puerto Rico assuming different timing of the lifting of NPIs. We estimated substantial heterogeneity in outcomes between states, that the timing of lifting NPIs is important, that even short delays in lifting could have a big impact, but that there is likely no amount of delay after which it would be completely safe to remove NPIs. Policy makers should consider the findings of this analysis as they monitor their state’s progress during the COVID-19 pandemic, project a suitable time to end restrictions, begin to discuss the conditions that must be met before declaring the pandemic over, and keep the public informed by making public health plans both safe and explicit. Ongoing vaccination efforts will help to contain the COVID-19 pandemic in 2022.

## Notes

Supplement.
eAppendix

eReferences
