# Supplementary material for: Projecting COVID-19 Mortality as States Relax Nonpharmacologic Interventions
Source: JAMA Health Forum. 2022 Apr 1;3(4):e220760. doi: 10.1001/jamahealthforum.2022.0760 (PMC8976243; doi:10.1001/jamahealthforum.2022.0760)
Supplement: Supplement. — eAppendix eReferences [file jamahealthforum-e220760-s001.pdf]

Supplemental Online Content

Linas BP, Xiao J, Dalgic OO, et al. Projecting COVID-19 mortality as states relax nonpharmacologic interventions. *JAMA Health Forum*. 2022;3(4):e220760. doi:10.1001/jamahealthforum.2022.0760

eAppendix

eReferences

This supplemental material has been provided by the authors to give readers additional information about their work.

© 2022 Linas BP et al. *JAMA Health Forum.*

eAppendix.

A1. Introduction

The *COVID-19 Policy Simulator* uses a mathematical model to simulate the COVID-19 pandemic at the national and state levels in the United States. The model is calibrated to historical trends in daily incident deaths and updated weekly as new data arises and the pandemic situation evolves. The online tool allows users to simulate the disease trajectory under different non-pharmaceutical interventions (NPIs) with varying timing and intensity. Forecasts are made for total cases, diagnosed cases, active cases, deaths, hospital bed occupancy, and ICU bed occupancy. Since May 2020, we have contributed weekly to the Centers for Disease Control and Prevention (CDC) COVID-19 Forecast Hub.[1]

A2. Development Timeline

eTable 1 lists major model updates and the dates on which they were introduced.

eTable 1. Major model updates.

Date	Update
February 2021	Vaccine rollout
August 2021	Age-stratification to incorporate age-stratified vaccine data and differential mortality of age groups
October 2021	Lower vaccine effectiveness due to Delta variant from August 1, 2021
December 2021	Waning (natural and vaccine-conferred) immunity
January 2022	Lower vaccine effectiveness due to Omicron variant from December 1, 2021

A3. Model Overview

Our model is an extension of the traditional susceptible-infected-recovered (SIR) model,[2] which partitions a population into compartments representing mutually exclusive disease states. At any time t, the variables $S(t)$, $E(t)$, $I(t)$, $R(t)$, and $D(t)$ denote the number of people in the susceptible, exposed, infected, recovered, and deceased compartments respectively. The flow of people between compartments is assumed to obey a system of deterministic ordinary differential equations. We let $\Delta t = 1$ to be compatible with data sources reporting daily data.

Age stratification

We stratify the population into two age groups, <65 years (low-risk) and ≥65 years (high-risk), with the subscript $a \in \{L, H\}$. The total population in age group a, denoted by N_a, is assumed to be constant over the simulation period.

Vaccination

To reflect administration guidelines of the Pfizer-BioNTech and Moderna vaccines,[i] we stratify the disease states by vaccination status. The subscript $v \in \{0, 1, 2\}$ denote the number of vaccine doses received under the recommended two-dose regime. The third vaccine, Janssen, approved for a single-dose regime, is omitted from the model due to its accounting for only 3.7% of all administered doses in the U.S. as of October 31, 2021.[ii] Since there is no data on vaccination status at the time of infection, we assume doses are allocated proportionally to the susceptible and recovered compartments over the

© 2022 Linas BP et al. *JAMA Health Forum.*

historical time horizon,[iii] i.e., if $V_{a,1}(t)$ and $V_{a,2}(t)$ are the actual number of first and second doses administered to age group a on day t, the proportion of the v-dose susceptible and recovered compartments, $v \in \{0, 1\}$, moving into the corresponding $(v+1)$-dose compartments on day t is

$$\alpha_{a,v+1}(t) = \min\left\{1, \frac{V_{a,v+1}(t-12)}{S_{a,v}(t) + R_{a,v}(t)}\right\}.$$

The time lag of 12 days accounts for the delay between receiving a vaccine dose and the beginning of protection.[3] The implicit assumption is that a susceptible person does not become infected in the 12 days after receiving a dose. The vaccine reduces both susceptibility to infection and mortality risk. After v vaccine doses, the probability of contracting the virus is reduced by $100 \times e_v^I\%$, with $0 = e_0^I \leq e_1^I, e_2^I \leq 1$; similarly, the infection fatality rate is reduced by $100 \times e_v^D\%$, with $0 = e_0^D \leq e_1^D, e_2^D \leq 1$.

Transmission

For a susceptible individual in age group a who has received v vaccine doses, the rate of exposure to the virus is given by

$$\lambda_{a.v} = (1 - e_v^I)\mathcal{R}(t)\gamma \sum_{a' \in \{L,H\}} c_{a,a'} \frac{I_{a',0}(t) + I_{a',1}(t) + I_{a',2}(t)}{N_{a'}},$$

where $\mathcal{R}(t)$ is the time-varying effective reproduction number. We model $\mathcal{R}(t)$ as a step function with breakpoints at the beginning of each calendar month over the historical time horizon to capture the effect of NPIs enforced during this period. The coefficients $c_{a,a'}$ are the elements of the contact matrix with row sums normalized to 1, so that $c_{a,a'}$ is the proportion of contacts per day of age group a that are with age group a'. When a susceptible individual contracts the virus, they enter the exposed state and remain there for the duration of the latent period with a mean of $1/\kappa$ days. After that, they transition to the infected state and remain there for the duration of the infectious period with a mean of $1/\gamma$ days. Finally, the infected individual will either die with probability $\delta_{a,v} = (1 - e_v^D)\delta_a$, where δ_a is the baseline infection fatality rate for age group a, or recover with probability $(1 - \delta_{a,v})$.

Waning immunity

An individual who has recovered from natural infection ($R_{a,v}$) enjoys a period of natural immunity with a mean of $1/\omega_n$ days before transitioning back into the susceptible state ($S_{a,v}$). A fully vaccinated susceptible individual ($S_{a,2}$) is protected for the duration of vaccine-conferred immunity with a mean of $1/w_v$ days before transitioning back into the partially susceptible state ($S_{a,1}$). Finally, since individuals with natural immunity who are subsequently vaccinated have been reported to exhibit "unusually potent immune responses",[4] a fully vaccinated recovered individual ($R_{a,2}$) is assumed to possess two 'layers' of immunity, shedding first their natural immunity then their vaccine-conferred immunity.

Booster shots

It is assumed that, once vaccinated, an individual will never shed their immunity completely (within the time frame of the simulation), and a fully vaccinated individual who has shed their vaccine-conferred

© 2022 Linas BP et al. *JAMA Health Forum.*

immunity is indistinguishable from a partially vaccinated individual. Thus, the model differentiates between the subpopulation that is willing to receive booster shots and the subpopulation that is unwilling to be vaccinated. Fully vaccinated individuals wane into the partially vaccinated state and are 'boosted' back into the fully vaccinated state.

Differential equation formulation

In summary, our model is described by the following system of equations, where (t) has been dropped for notational simplicity:

$$
\begin{aligned}
\dot{S}_{a,0} &= -(\lambda_{a,0} + \alpha_{a,1})S_{a,0} + \omega R_{a,0},\\
\dot{E}_{a,0} &= \lambda_{a,0}S_{a,0} - \kappa E_{a,0},\\
\dot{I}_{a,0} &= \kappa E_{a,0} - \gamma I_{a,0},\\
\dot{R}_{a,0} &= (1-\delta_{a,0})\gamma I_{a,0} - (\alpha_{a,1} + \omega)R_{a,0},
\end{aligned}
$$

$$
\begin{aligned}
\dot{S}_{a,1} &= -(\lambda_{a,1} + \alpha_{a,2} + \alpha_{a,3})S_{a,1} + \alpha_{a,1}S_{a,0} + \omega(R_{a,1} + \alpha'_{a,3}S_{a,2}),\\
\dot{E}_{a,1} &= \lambda_{a,1}S_{a,1} - \kappa E_{a,1},\\
\dot{I}_{a,1} &= \kappa E_{a,1} - \gamma I_{a,1},\\
\dot{R}_{a,1} &= (1-\delta_{a,1})\gamma I_{a,1} + \alpha_{a,1}R_{a,0} - (\alpha_{a,2} + \alpha_{a,3} + \omega)R_{a,1},
\end{aligned}
$$

$$
\begin{aligned}
\dot{S}_{a,2} &= -\lambda_{a,2}S_{a,2} + (\alpha_{a,2} + \alpha_{a,3})S_{a,1} + \omega\alpha'_{a,3}(R_{a,2} - S_{a,2}),\\
\dot{E}_{a,2} &= \lambda_{a,2}S_{a,2} - \kappa E_{a,2},\\
\dot{I}_{a,2} &= \kappa E_{a,2} - \gamma I_{a,2},\\
\dot{R}_{a,2} &= (1-\delta_{a,2})\gamma I_{a,2} + (\alpha_{a,2} + \alpha_{a,3})R_{a,1} - \omega\alpha'_{a,3}R_{a,2},
\end{aligned}
$$

$$
\dot{D} = \delta_{a,0}\gamma I_{a,0} + \delta_{a,1}\gamma I_{a,1} + \delta_{a,2}\gamma I_{a,2}.
$$

The initial conditions are $\left(I_{a,0}(0),\ S_{a,0}(0)\right) = \left(I_{a,0}^{\text{init}},\ N_a - I_{a,0}^{\text{init}}\right)$ and zero for all other variables. eTables 2 and 3 display the values or ranges for all model parameters and their references.

eTable 2. Estimates of fixed and calibrated parameters.

Parameter	Estimate	Reference and notes
Fixed parameters		
N_L	State-dependent	U.S. Census Bureau: SC-EST2020-AGESEX-CIV: POPEST2019_CIV
N_H		
$[c_{L,L}, c_{L,H}; c_{H,L}, c_{H,H}]$	[0.93, 0.07; 0.48, 0.52]	[5] Aggregate columns and rows into age groups <65 years and ≥65 years, then normalize so that rows sum to 1.
κ	1/5.5	[6]
γ	1/10	[7]
ω_n, w_v	16 months	[8]

© 2022 Linas BP et al. *JAMA Health Forum.*

		Reinfection by endemic SARS-CoV-2 is expected to occur between 3 months and 5 years after peak antibody response, with a median of 16 months.
Calibrated parameters		
$\mathcal{R}(t)$	0.5–6.0	[9, 10, 11, 12] Widely varying by location and SARS-CoV-2 variant.
I^{init}	100–10,000	Divided proportionally into: • $I_L^{\text{init}} = \frac{N_L}{N_L+N_H} I^{\text{init}}$, • $I_H^{\text{init}} = \frac{N_H}{N_L+N_H} I^{\text{init}}$.

eTable 3. Evolution of estimates of variant-dependent parameters.

Parameter	Estimate	Reference and notes
Baseline values		
δ_L	0.001	Based on this meta-analysis. These values chosen to approximate the CDC's "Estimated Total Infections".
δ_H	0.030	
$1 - e_1^I$	0.54	[13] Table 2: "Documented Infection" at "14 to 20 days after first dose": $(1 - \text{RR})\% = 46$. Derivation: $1 - e_1^I = 1 - 0.46$.
$1 - e_2^I$	0.08	[13] Table 2: "Documented Infection" at "7 days after second dose to end of follow-up": $(1 - \text{RR})\% = 92$. Derivation: $1 - e_2^I = 1 - 0.92$.
$1 - e_1^D$	0.52	[13] Table 2: "Death" at "14 to 20 days after first dose": $(1 - \text{RR})\% = 72$. Derivation: $(1 - e_1^I)(1 - e_1^D) = 1 - 0.72$.
$1 - e_2^D$	0.63	Vaccine clinical trials report 95% efficacy against death. Derivation: $(1 - e_2^I)(1 - e_2^D) = 1 - 0.95$. Note that it is the *conditional probability* of death that is higher after the second dose than after the first dose. If a fully vaccinated individual contracts a breakthrough infection despite 92% reduction in susceptibility, it is plausible that they are particularly vulnerable and have a smaller reduction in mortality risk *conditional on infection* compared to a partially vaccinated individual who contracts a breakthrough infection.
Delta variant (parameter values change at a linear rate as the variant saturates over August 2021)		
$1 - e_2^I$	0.13	[14] "Two dose vaccine effectiveness was 86.7% (95% confidence interval 84.3% to 88.7%) against infection with the delta variant, ..." Derivation: $(1 - e_2^I) = 1 - 0.87$.
$1 - e_1^I$	0.90	Scaled up proportionally to e_2^I. Derivation: $1 - e_1^I = 0.54 \times (0.13/0.08)$.
δ_L	0.0023	[15] "Increased risk with the Delta variant was more pronounced at ... 133% (95% CI 54%–231%) for death." Derivation: $\delta_L = 0.001 \times 2.33$. Derivation: $\delta_H = 0.030 \times 2.33$.
δ_H	0.0700	

© 2022 Linas BP et al. *JAMA Health Forum.*

		We assume that the increased mortality of Delta is a consequence of higher IFR only, not lower vaccine effectiveness against death after infection.
Omicron variant (parameter values change at a linear rate as the variant saturates over December 2021)		
$1 - e_2^I$	0.30	UKHSA Report: Figure 2B
δ_L	0.0008	UKHSA Report: "The risk of hospital admission from emergency departments with Omicron was approximately one-third of that for Delta (Hazard Ratio 0.33, 95% CI: 0.30 to 0.37)."
δ_H	0.0233	Same as above.

A4. Calibration and Numerical Solution

We calibrate the model to historical daily incident deaths. The system of ordinary differential equations is solved numerically using Euler's method (R package `deSolve`).[16] The calibration method is generalized simulated annealing (R package `GenSA`) with the sum of squared errors as the objective function.[17] To account for uncertainty in the calibrated values, we repeat the calibration process 100 times with different initial solutions, resulting in 100 unique sets of parameter values and fitted curves. At each time point, we take the median as the point estimate and compute the 90% coverage simulation band.

eTable 4 displays our input data and their references.

eTable 4. Input data.

Data	Source	Reference
COVID-19 cases and deaths	JHU CSSE	[18]
Vaccine administration	Our World in Data and CDC	[19] and CDC

A5. Forecasting

We make forecasts by allowing the model to continue running past the historical time horizon. Diagnosed cases and hospital and ICU occupancy are not accounted for in the SEIR model. We estimate these in a post-processing step as follows.

Diagnosed cases
We assume the future diagnosis rate remains at the latest estimated value, i.e., the number of incident diagnosed cases on the last day of data divided by the number of incident total cases on the last day of data.

Hospital and ICU bed occupancy
We back-calculate hospital and ICU bed occupancy from incident deaths assuming an average time to death from hospital and ICU admission of 16 and 10 days respectively.[20] Starting in August, we forecast occupancy data provided by the U.S. Department of Health and Human Services. Note that the data does not include all hospitals in any given state so our forecasts do not estimate the total demand for hospital and ICU beds.

A6. Non-Pharmaceutical Interventions

© 2022 Linas BP et al. *JAMA Health Forum.*

The *Simulator* offers four types of non-pharmaceutical interventions:

1. *Current interventions:* The effective reproduction number remains at the latest calibrated value.
2. *Lockdown:* The effective reproduction number is 0.3, the estimated value in Wuhan during the strict lockdown of the city starting in March 2020.[21]
3. *Stay-at-home orders:* The effective reproduction number is the lowest calibrated value attained during the period from March to July 2020.
4. *Minimal restrictions:* The effective reproduction number is the basic reproduction number, which changes with the proportions of the circulating variants of concern.

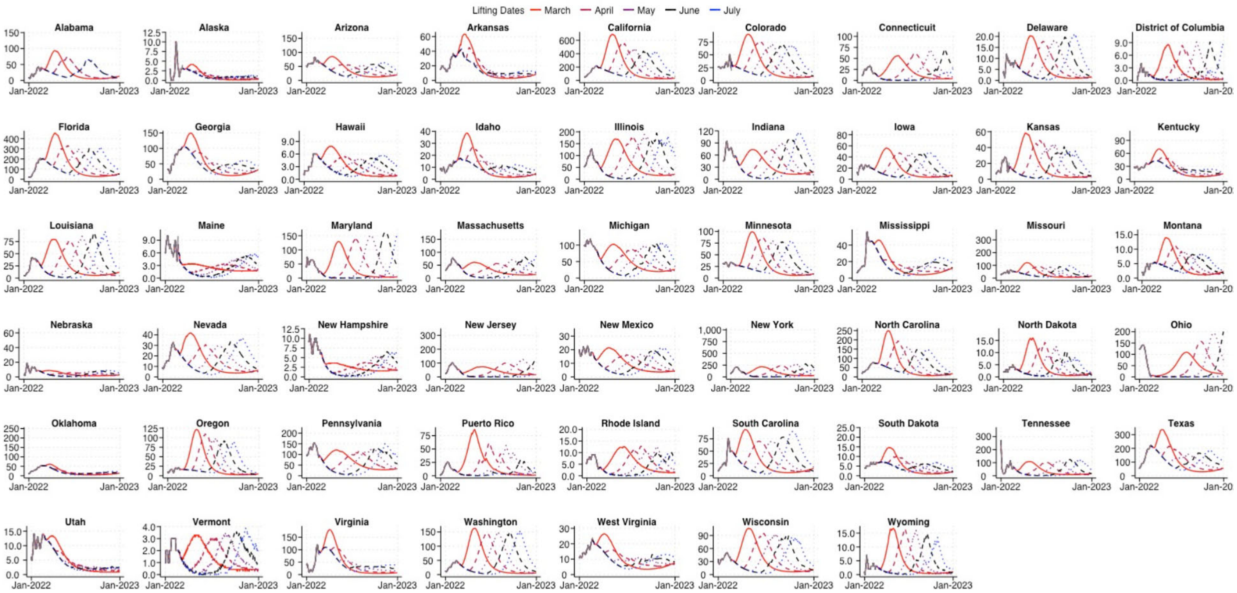

(a)

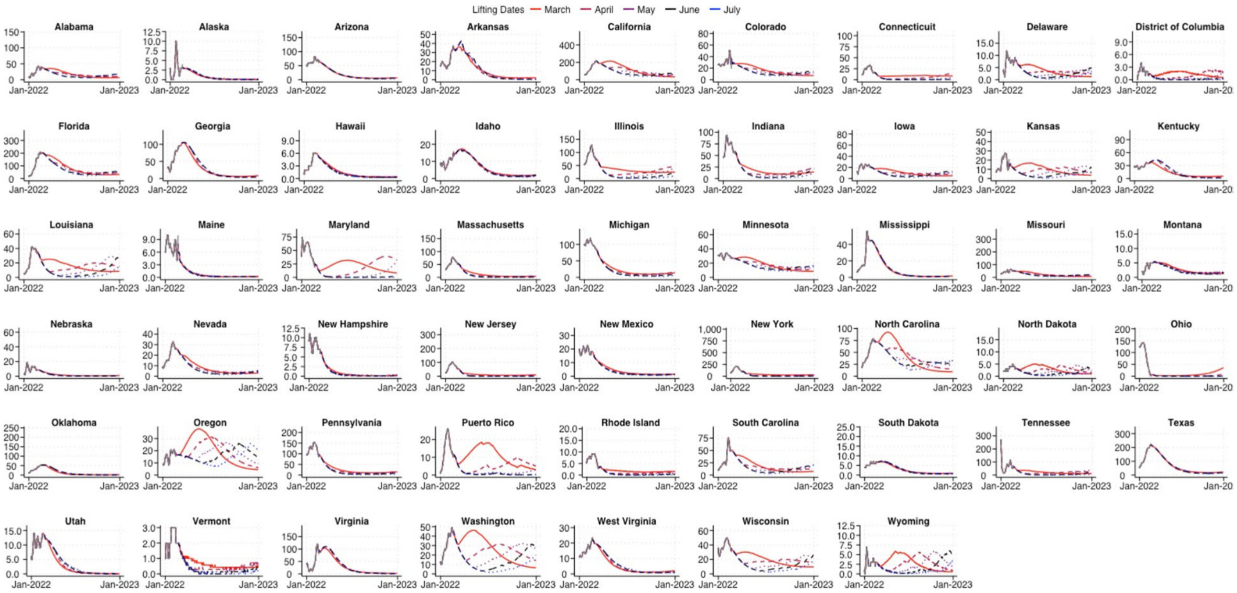

(b)

eFigure 1. Model-based projections of COVID-19 deaths following the lifting of NPIs in each state, assuming an effective reproduction number of 5.0 (eFigure 1a) and 3.0 (eFigure 1b).

© 2022 Linas BP et al. *JAMA Health Forum.*

eReferences

1. Cramer, E. Y. *et al.* Evaluation of individual and ensemble probabilistic forecasts of COVID-19 mortality in the US. *medRxiv* 2021.02.03.21250974 (2021) doi:10.1101/2021.02.03.21250974.
2. Kermack, W. O., McKendrick, A. G. & Walker, G. T. A contribution to the mathematical theory of epidemics. *Proc. R. Soc. Lond. Ser. Contain. Pap. Math. Phys. Character* **115**, 700–721 (1927).
3. Mahase, E. Covid-19: Pfizer vaccine efficacy was 52% after first dose and 95% after second dose, paper shows. *BMJ* **371**, m4826 (2020).
4. Crotty, S. Hybrid immunity. *Science* **372**, 1392–1393 (2021).
5. Prem, K., Cook, A. R. & Jit, M. Projecting social contact matrices in 152 countries using contact surveys and demographic data. *PLOS Comput. Biol.* **13**, e1005697 (2017).
6. Xin, H. *et al.* Estimating the Latent Period of Coronavirus Disease 2019 (COVID-19). *Clin. Infect. Dis.* (2021) doi:10.1093/cid/ciab746.
7. Byrne, A. W. *et al.* Inferred duration of infectious period of SARS-CoV-2: rapid scoping review and analysis of available evidence for asymptomatic and symptomatic COVID-19 cases. *BMJ Open* **10**, e039856 (2020).
8. Townsend, J. P. *et al.* The durability of immunity against reinfection by SARS-CoV-2: a comparative evolutionary study. *Lancet Microbe* (2021) doi:10.1016/S2666-5247(21)00219-6.
9. Hilton, J. & Keeling, M. J. Estimation of country-level basic reproductive ratios for novel Coronavirus (SARS-CoV-2/COVID-19) using synthetic contact matrices. *PLOS Comput. Biol.* **16**, e1008031 (2020).
10. Billah, M. A., Miah, M. M. & Khan, M. N. Reproductive number of coronavirus: A systematic review and meta-analysis based on global level evidence. *PLOS ONE* **15**, e0242128 (2020).
11. Ke, R., Romero-Severson, E., Sanche, S. & Hengartner, N. Estimating the reproductive number R0 of SARS-CoV-2 in the United States and eight European countries and implications for vaccination. *J. Theor. Biol.* **517**, 110621 (2021).
12. Liu, Y. & Rocklöv, J. The reproductive number of the Delta variant of SARS-CoV-2 is far higher compared to the ancestral SARS-CoV-2 virus. *J. Travel Med.* **28**, (2021).
13. Dagan, N. *et al.* BNT162b2 mRNA Covid-19 Vaccine in a Nationwide Mass Vaccination Setting. *N. Engl. J. Med.* **384**, 1412–1423 (2021).
14. Bruxvoort, K. J. *et al.* Effectiveness of mRNA-1273 against delta, mu, and other emerging variants of SARS-CoV-2: test negative case-control study. *BMJ* **375**, e068848 (2021).
15. Fisman, D. N. & Tuite, A. R. Evaluation of the relative virulence of novel SARS-CoV-2 variants: a retrospective cohort study in Ontario, Canada. *CMAJ Can. Med. Assoc. J. J. Assoc. Medicale Can.* **193**, E1619–E1625 (2021).
16. Soetaert, K., Petzoldt, T. & Setzer, R. W. Package deSolve: Solving Initial Value Differential Equations in R. 52.
17. Xiang, Y., Gubian, S., Suomela, B. & Hoeng, J. Generalized Simulated Annealing for Global Optimization: The GenSA Package. *R J.* **5**, 13 (2013).
18. Dong, E., Du, H. & Gardner, L. An interactive web-based dashboard to track COVID-19 in real time. *Lancet Infect. Dis.* **20**, 533–534 (2020).
19. Mathieu, E. *et al.* A global database of COVID-19 vaccinations. *Nat. Hum. Behav.* 1–7 (2021) doi:10.1038/s41562-021-01122-8.

© 2022 Linas BP et al. *JAMA Health Forum.*

20. Ferguson, N. *et al. Report 9: Impact of non-pharmaceutical interventions (NPIs) to reduce COVID19 mortality and healthcare demand. 20* http://spiral.imperial.ac.uk/handle/10044/1/77482 (2020) doi:10.25561/77482.
21. Pan, A. *et al.* Association of Public Health Interventions With the Epidemiology of the COVID-19 Outbreak in Wuhan, China. *JAMA* **323**, 1915–1923 (2020).

---

[i] See https://www.cdc.gov/coronavirus/2019-ncov/vaccines/second-shot.html for details.
[ii] See https://ourworldindata.org/grapher/covid-vaccine-doses-by-manufacturer?country=~USA for details.
[iii] The CDC advises against vaccination while under active infection. See https://www.cdc.gov/coronavirus/ 2019-ncov/vaccines/faq.html for details.

© 2022 Linas BP et al. *JAMA Health Forum.*
